# Meta-analysis unravels common responses of seed oil fatty acids to temperature for a wide set of genotypes of different plant species

**DOI:** 10.3389/fpls.2024.1476311

**Published:** 2024-11-15

**Authors:** Constanza Alberio, Luis A. N. Aguirrezábal

**Affiliations:** ^1^ Instituto de Innovación para el Desarrollo Agroalimentario y Agroenergético Sostenible (IIDEAGROS), Laboratorio de Fisiología Vegetal, Facultad de Ciencias Agrarias, Universidad Nacional de Mar del Plata (FCA-UNMdP), Balcarce, Argentina; ^2^ Consejo Nacional de Investigaciones Científicas y Técnicas, Buenos Aires, Argentina

**Keywords:** oil quality modeling, metaphenomics, oil fatty acid response, genotypic variation in fatty acids, oilseeds

## Abstract

Temperature is the main environmental determinant of seed oil fatty acid Q9 composition. There are no models describing common responses of main seed oil fatty acids to temperature in plants. The aim of thus work was to investigate common responses of seed oil fatty acids to minimum temperature during grain filling across species and genotypes. A database consisted of 164 genotypes of 9 species, sunflower, rapeseed, soybean, maize, flax, chia, safflower, olive and camelia, grown under a wide range of environmental conditions, was created and analyzed applying meta phenomics tools. Four widely sown species of the database was used to develop several common seed fatty acid responses and validate some models, and the other species were used to validate the General Model. The minimum temperature during grain filling responses of fatty acids in the General Model were close to responses found in genotypes of five independent species used to validate the model. Dissections of the general model by selecting the appropriate data allowed unraveling previously unknown features of the response of fatty acid to the minimum temperature during grain filling. The response of fatty acids to temperature for any species was unaffected by experimental conditions (field or controlled conditions) during the oil synthesis stage. The oleic acid trait did not affect the response to temperature of fatty acids synthesized downstream and upstream of it. Traits such as high stearic or high linoleic did not affect the response of fatty acids synthesized upstream or downstream of the trait. The established models and new knowledge could be applied to design cost effective and timely experiments to assess the potential responses of seed oil fatty acids to temperature of previously untested genotypes.

## 1 Introduction

It has long been recognized that temperature is the primary environmental factor influencing the composition of seed oil fatty acids of different crops (Canvin, 1965), affecting the final seed oil quality. Seeds are not only the harvest organ for industrial use, but also a reserve and perpetuation organ of the species (Izquierdo et al., 2017), whose oil is consumed by humans or used as a sub-product for biofuel. Literature documents the responses of fatty acids to minimum temperature during grain filling of seeds across various crops as well as different genotypes of these crops (Aguirrezábal et al., 2009, 2015 and references therein). However, this fragmented information reveals significant variability in the response of oil fatty acids to minimum temperature during seed filling, both among and within species and genotypes (Zanetti et al., 2022; Brandán et al., 2022; Abou Chehade et al., 2022; Angeloni et al., 2017; Baux et al., 2013; Zuil et al., 2012). Thus, a model encompassing common responses of fatty acids to minimum temperature from seed oils across a wide range of species and genotypes has not yet been established.

The absence of such a model likely stems from experimental challenges, including: i) conducting simultaneous experiments with species of diverse life forms and architectural traits; ii) synchronizing the grain filling period among species and genotypes that vary in their thermal and photoperiodic requirements for development, as well as their thermal and radiation needs for growth; iii) integrating results obtained under controlled conditions with those from field studies. Combining data from plants cultivated under both conditions is contentious due to significant abiotic factors that alter the growing environment (see Poorter et al., 2012, 2016 and references therein).

Given these difficulties, conducting experiments with a broad array of genotypes and species in field conditions, under varying minimum temperatures during grain filling [e.g., across multiple locations, latitudes, sowing dates, or by manipulating temperatures in the same field (Izquierdo et al., 2002; García-Inza et al., 2014, 2018)], is difficult. Similarly, adjusting temperatures during grain filling in growth chambers or greenhouses (Izquierdo et al., 2006, 2008) requires extensive experimental efforts and significant human and technical resources. These efforts are still necessary to concurrently cultivate numerous genotypes of different species, particularly when utilizing large-scale phenotyping facilities (e.g., Minervini et al., 2017).

The high intraspecific variability in the response of seed oil fatty acid composition to temperature may be attributed to natural variation and genetic enhancement. Various genotypes of multiple oil-producing species, which exhibit high percentages of specific fatty acids, have been developed for a range of applications. For example, high oleic (HO) genotypes have been developed in several species, significantly increasing the oleic acid content compared to traditional genotypes. E.g., sunflower (Soldatov, 1976): ~75% *vs* ~35%; rapeseed (Alberio, 2017): ~81% *vs* ~60%; soybean (Kinney and Knowlton, 1998): ~84% *vs* ~17%; maize (Beló et al., 2008): ~39% *vs* ~25%; safflower (Zemour et al., 2021; Zanetti et al., 2022): ~75% *vs* ~12%. It is generally accepted that the response of fatty acids to minimum temperature in HO genotypes is lower compared to traditional (wild type, WT) genotypes (Zuil et al., 2012; Alberio et al., 2016; Alberio, 2017, unpublished data). However, some studies report similar responses between certain HO genotypes and WT genotypes (Angeloni et al., 2017; Izquierdo et al., 2009). Furthermore, the response of oil fatty acids to minimum temperature can be modified by the genetic background in which HO mutations are present (Alberio et al., 2018).

Conversely, genotypes with mutations other than HO have also been obtained in several species [e.g., high stearic (Fernández-Moyá et al., 2002) and low linolenic (Deng and Scarth, 1998)]. Additionally, genotypes combining mutations in two genes of the fatty acid synthesis pathways, coding for different fatty acids, have been developed. These genotypes enhance the percentage of two fatty acids in the seed oil [e.g., high palmitic/high linoleic in sunflower (Osorio et al., 1995), high palmitic/high oleic in sunflower (Fernández-Martínez et al., 1997), high oleic/low linolenic in rapeseed (Scarth and McVetty, 1999)]. The response of fatty acids to minimum temperature in genotypes carrying these traits or their combinations is not well understood (Izquierdo et al., 2013). It remains unclear whether these traits affect the responses of other fatty acids that are not directly influenced by these mutations (e.g., upstream or downstream in the synthesis pathway).

Meta-analytical approaches can be employed to combine results from various studies and unpublished data into comprehensive response models (e.g., Poorter et al., 2010). These approaches provide insights into the phenotypic plasticity [i.e., a plant’s ability to respond to changes in resource availability (Bradshaw, 1965)] across a range of genotypes. Meta-analysis has not been previously applied to explore common response patterns of fatty acids to temperature across numerous species and genotypes within species. This approach could be instrumental in dealing the high intraspecific variability in the percentage of the same fatty acid among species and even among genotypes within the same species.

Therefore, the aim of this study was to investigate common responses of seed oil fatty acids to minimum temperature during grain filling across species and genotypes. This was achieved by conducting meta-analysis using a purpose-built database comprising a wide set of genotypes from nine species grown under varied environmental conditions. To achieve this objective, we first established a General Model by identifying common responses of main seed oil fatty acids to minimum temperature during grain filling in genotypes of four widely cultivated crop species (sunflower, soybean, rapeseed and maize). By selecting appropriate datasets, specific responses were identified: i) interspecific responses of different fatty acids to minimum temperature during grain filling within these four species, ii) effects of the HO trait on these responses, and iii) behavior of fatty acids when cultivating plants in the field or under controlled conditions in pots. Finally, we assessed the scope of the established models using independent data of: iv) genotypes carrying mutations other than HO in the oil fatty acid synthesis pathway, and v) species whose responses of fatty acids to temperature have been less investigated in prior research.

## 2 Materials and methods

### 2.1 Database

A database was compiled from 48 sources, both published and unpublished, documenting seed oil fatty acid compositions in relation to minimum temperature during grain filling across 160 genotypes from nine species (totaling 5406 data pairs of fatty acids *vs*. minimum temperature). Data were sourced by i) extracting information from tables or graphs in articles presenting both temperature data during grain filling and fatty acid compositions of seed oils, and ii) incorporating unpublished results from our laboratory (e.g., Alberio, 2017). All species included in the database exhibited seed oil compositions of palmitic, stearic, oleic, linoleic, and linolenic acids, except for sunflower, whose seed oil typically contains only trace amounts of linolenic acid. To achieve the objectives of this study, the database was divided into three distinct datasets, designated as DataSet A, B, and C.

DataSet A (**Table 1**) was utilized to establish a General Model of fatty acid responses to temperature. This dataset comprised 122 genotypes from four species: sunflower (SF, 50.8% of DataSet A), rapeseed (R - 32.7%), soybean (S - 13.1%), and maize (M - 3.2%). These species were chosen due to their widespread cultivation and the availability of published and unpublished fatty acid data related to temperature. DataSet A was further subdivided to develop three additional models. Firstly, to explore interspecific differences in fatty acid responses to minimum temperature, the dataset was split into sunflower data (SF-model) and combined data from rapeseed, soybean, and maize (RSM-model). Secondly, to investigate the impact of growth conditions on fatty acid responses, data were split based on whether plants were grown under field conditions or controlled environments. Finally, the influence of the high oleic (HO) trait was examined by establishing separate responses for genotypes with traditional (wild type, WT) and high oleic profiles. DataSet A included 46 genotypes with the HO trait and 76 WT genotypes.

**Table 1:** Description of DataSet A used for developing the General Model and dissections of the response of fatty acids seed oil to temperature.

Species	Number of genotypes	Traits	Source	Temperature range (°C)
Sunflower	9	WT/HO/UHO	Alberio et al., 2018	13.3-22.3
	3	WT/HO/UHO	Alberio et al., 2016	11.8-23.2
	8	WT	Muratorio, 2003	17.1-23.1
	1	WT	Canvin, 1965	8.7-26.1
	1	WT	Tremolieres et al., 1982	12.0-27.0
	1	WT	Izquierdo et al., 2006	10.2-26.2
	1	WT	Echarte et al., 2010	15.2-22.3
	8	WT/HO	Izquierdo and Aguirrezábal, 2008	12.5-21.5
	2	WT/HO	Zuil and Aguirrezabal (not published data)	14.2-16.8
	13	HO	Angeloni et al., 2017	12.9-21.6
	1	WT	Fernández-Moyá et al., 2002	10.0-25.0
	2	WT/HO	Martínez-Force et al., 1999	15.0
	8	WT	Werteker et al., 2010	16.3-18.7
	2	HO	Regitano Neto et al., 2016	10.8-18.3
	2	WT/HO	Izquierdo et al., 2013	16.0-26.0
Rapeseed	5	WT	Aslam et al., 2009	8.1-10.4
	1	WT	Canvin, 1965	13.2-25.6
	1	WT	Tremolieres et al., 1982	12.0-27.0
	6	WT	Agosti, 2011	13.4-16.1
	8	WT	Baux et al., 2013	9.6-16.4
	7	WT/HO	Alberio, 2017	11.8-15.7
	1	WT	Deng and Scarth, 1998	10.0-25.0
	10	WT	Werteker et al., 2010	13.1-25.4
	1	WT	Schulte et al., 2013	18.1-25.3
Soybean	2	WT/HO	Izquierdo et al., 2009	12.9-25.1
	2	WT/HO	Zuil et al., 2012	9.2-14.9
	1	WT	Schulte et al., 2013	21-5-25.9
	9	WT	Werteker et al., 2010	12.1-16.9
	1	WT	Garces et al., 1992	10.0-20.0
	1	HO	Rebetzke et al., 1996	17.6-26.6
Maize	2	WT/HO	Izquierdo et al., 2009	13.0-25.1
	2	WT/HO	Zuil et al., 2012	13.0-20.9

This table includes species, the number of genotypes involved in each experiment, traits associated with at least one genotype, data sources and the range of temperature explored by each experiment. WT, wild-type (traditional) genotypes; HO, high oleic genotypes. The dataset comprises a total of 5,026 pairs of data points (fatty acid *vs*. temperature).

The validity of models developed using DataSet A was assessed using DataSet B and DataSet C. DataSet B (**Table 2**) comprised 12 genotypes, each carrying at least one trait affecting fatty acid composition (HS: high stearic, HP: high palmitic, HL: high linoleic, and LL: low linolenic), different from the HO trait. In most cases, these genotypes also included the HO trait. For sunflower, tested genotypes included HS, HP, HSHL, and HSHO; for rapeseed, LL and HOLL genotypes were tested. Detailed information on studied mutations in the fatty acid synthesis pathway was available for sunflower and rapeseed (**Supplementary Material**). DataSet C (**Table 3**) consisted of 32 genotypes from Camellia (34.6% of DataSet C), Safflower (26.9%), Chia (15.4%), Flax (15.4%), and Olive (7.7%), some of which included WT, HO, and HL genotypes (see **Table 3** for details).

**Table 2:** Description of DataSet B, used to test the validity domain of the General Model and its dissections.

Species	Number of genotypes	Traits	Source	Temperature range (°C)
Sunflower	4	HSHL/HSHO	Alberio, 2017	12.8-21.6
	1	HS	Fernández-Moyá et al., 2002	10.0-24.0
	2	HPHL/HPHO	Martínez-Force et al., 1999	15.0
	2	HSHL/HSHO	Izquierdo et al., 2013	15.0
Rapeseed	2	HOLL	Baux et al., 2013	11.1-17.4
	1	LL	Deng and Scarth, 1998	10.0-25.0

This dataset includes genotypes with traits that modify fatty acid composition differently from the HO trait. The table provides details on species, the number of genotypes included in each experiment, and the specific traits of the genotypes: HSHL, high stearic/high linoleic; HSHO, high stearic/high oleic; HOLL, high oleic/low linolenic; LL, low linolenic, as well as the data source and the range of temperature explored by each experiment. The total dataset comprises 257 pairs of data points (fatty acid *vs*. temperature).

**Table 3:** Description of DataSet C, used to test the validity domain of the established General Models and their sub-models.

Species	N°	Carrying mutation	Source	Temperature range (°C)
Flax	1	WT	Canvin, 1965	10.0-25.8
	3	WT	Green, 1986	20.0
	1	WT	Dybing and Zimmerman, 1966	15.0-25.0
Chia	1	WT	Mack et al., 2018	15.7
	1	WT	Ayerza, 1995	10.3-14.3
	2	WT	Brandán et al., 2022	11.5-12.1
Safflower	6	HO/HL	Abou Chehade et al., 2022	16.8-16.9
	3	WT	Zemour et al., 2021	24.1-25.2
	1	HO	Zanetti et al., 2022	8.7-13.4
	1	WT	Canvin, 1965	12.3-26.1
Olive	1	WT	Garcia-Inza et al., 2018	16.6-26.5
	1	WT	Mafrica et al., 2021	14.4-17.7
Camelia	9	WT	Zanetti et al., 2017	7.9-11.7

This dataset includes species not used in the model development. The table provides details on the species, the number of genotypes, the traits of the genotypes, the data source and the range of temperature explored by each experiment. The dataset comprises a total of 123 pairs of data points (fatty acid *vs*. temperature).

The average minimum temperature during fruit filling was used in this work as the input variable of the response of all fatty acid to temperature. This expression of temperature was selected because it was the best predictor of oleic and linoleic acids in sunflower oil seed (Izquierdo et al., 2002 in WT and HO genotypes, Angeloni et al., 2017 in medium and HO genotypes), and of oleic, linoleic and linolenic acids in rape oil seed (Baux et al., 2008, 2013 in WT, LL and HOLL genotypes). Also, in soybean and maize the average minimum temperatures performed as well as the medium temperatures as predictors of those fatty acids in the seed oil (Izquierdo et al., 2009). At the best of our knowledge, precise studies about the best temperature predictor have not been performed neither for saturated fatty acids of sunflower, soybean, rapeseed and maize nor in other species.

Average minimum temperatures across the entire database ranged widely during grain filling (9.2 – 26.2°C). This variability arose from cultivation under controlled conditions or in diverse field environments, encompassing different locations, sowing dates, and seasons. On the other hand, this range of minimum temperatures is outside from the thresholds of extreme temperatures (both low and high). that cause damage in the set of studied species (e.g. Rife and Zeinali, 2003: Tetreault et al., 2016; Ahmad et al., 2021).

The output variable, corresponding to the percentage of each fatty acid in the oil, highly differed across species and genotypes within species. For instance, oil of WT differed between 40 and 72% between the analyzed species (Green, 1986; Garcés et al., 2009; Baux et al., 2013; Zanetti et al., 2017; García-Inza et al., 2016; Mack et al., 2018; Abou Chehade et al., 2022), while considering the oil of high oleic genotypes can reach up to 93% of oleic acid (e.g. sunflower [~93%], rapeseed [~83%]) (data extracted from database, **Tables 1**, **3**). These differences in percentages of a same fatty acid among species made not possible to establish single generalized response curves of fatty acids *vs*. temperature by using raw data. Thus, the output variable, data of fatty acid percentages, was normalized (see 2.2. “Phenotypic response model” subsection).

### 2.2 Phenotypic response models

#### 2.2.1 Establishment of the general model

The percentage of each fatty acid of each genotype was normalized following Poorter et al. (2010). The output variable was defined as the percentage of each fatty acid relativized to a reference value. The reference value for normalization was set at Tmin = 15.5°C for practical convenience. Data from all genotypes overlapped in the range of Tmin from 10 to 16°C while the median temperature (Tmin = 16.2°C) was outside of this range. Genotypes whose highest or lowest Tmin differed by ≥10% (<1.5°C) from Tmin = 15.5°C were excluded from further analysis.

An interpolation from the reference temperature of each genotype was performed when Tmin was different from 15.5°C during grain filling. A trend line was adjusted for experiments where the range of explored Tmin covered the reference temperature (98.3% of the whole dataset). Data were normalized as described in Equation 1.


(1) Fatty acid (scaled)=Fatty acid(%)/interpolated Fatty acid(%)

Available data were analyzed even when genotypes did not present information of every fatty acid. Genotypes presenting just one point of fatty acid percentage and Tmin (e.g. Martínez-Force et al., 1999) were not considered for analysis as it was not possible to normalize these values using the method above described. These isolated data were treated in a special way to include them in the splits of the DataSet A (see section 2.1).

The general phenotypic response of each fatty acid to average minimum temperature during grain filling was characterized using i) scatter plots and ii) reaction norms. **Supplementary Figure 1** provides examples of both graphical representations using raw versus normalized and interpolated data for oleic acid in genotypes from DataSet A:

i- Scatter plot: Visualizes data point distribution, indicating trends and genotype-specific variations within the dataset (**Supplementary Figure 2A**). The general trend, the distribution of data points from different species or genotypes responding distinctly within this cloud, and thus the variation of the genotype response tend with respect to the general trend (**Supplementary Figure 2A**).

ii- Reaction norm model: Describes phenotypic plasticity by depicting the shape and range of the phenotypic response to minimum temperature (the set of phenotypes that a genotype produces when exposed to different environmental conditions, **Supplementary Figure 2B**). Phenotypic plasticity is defined by the direction, magnitude, and extent of the reaction norm in response to environmental conditions (Arnold et al., 2019; Nicotra et al., 2010). A more detailed interpretation of reaction norms is provided in the **Supplementary Material**, specifically below **Supplementary Figure 2**.

Minimum temperature was categorized into seven 2°C intervals (8-10, 11-13, 14-16, 17-19, 20-22, 23-25, and 26-28°C) for reaction norm and scatter plot analysis. Phenotypic response was characterized by drawing the reaction norm including median, first and third interquartile ranges (Q25 and Q75), and 10% (P10) and 90% (P90) percentiles..

#### 2.2.2 Comparison of the response curves for plants grown in the field or under controlled conditions including or not the HO trait

The General Model established by using DataSet A (see section 2.1.) was further studied to analyze in detail groups of species or genotypes as well as growing conditions during the grain filling period (see section 1).

First, DataSet A was split considering data of sunflower on one side (SF) and the combined data of rapeseed, soybean and maize altogether (RSM) on the other. The linolenic acid is present in all species of DataSet A except in sunflower.

Second, the data were split for field and controlled conditions grown plants to investigate the influence of growing conditions during fruit filling on the response of fatty acids to temperature.

Finally, the phenotypic response of WT and HO genotypes included in the DataSet A (**Table 1**) were separately characterized. Thus, the DataSet A was divided into traditional (WT) and high oleic (HO) genotypes, independently of the species. WT and HO models were established following the same procedure to establish the General Model. Once these models were established, previously discarded isolated data (Section 2.2.1) were here included. This was performed by using the trend lines established for the WT or HO models (depending on the isolated data corresponded to a WT or HO genotype, respectively). Applying the method described in Section 2.2.1, isolated data were normalized to the reference Tmin value.

#### 2.2.3 Testing the validity of the domain of the established models

The validity domain of WT and HO models to account for the response of fatty acids to Tmin was tested using the set of genotypes included in DataSet B. This DataSet included genotypes carrying at least one trait modifying the fatty acid composition, different from the HO trait. In most of cases, the genotypes also carried the HO trait. Data of DataSet B were transformed and treated as described in Section 2.2.1. The transformed data of DataSet B were superimposed on the relationships of the established WT model or the HO model, as appropriate. When superimposed data were within the range of prediction line of each model (WT or HO models) it was considered that they were within the validity domain of the tested model. Fittings of DataSet B were established. Slopes and ordinates were determined and compared with those of WT or HO models.

The validity domain of General Model, SF-Model and RSM-Model were tested as described in the precedent paragraph by using genotypes of species included in DataSet C (**Table 3**). The species that made up the DataSet C were independent than those used to establish the different models. DataSet C included data from flax, chia, camellia, safflower and olive (seed and mesocarp). In all genotypes the oil is mainly accumulated in the seeds except in olive where the oil is mostly cumulated in the mesocarp. Genotypes further tested were those whose ranges of Tmin were ≤ 2°C (Tmin intervals = 2°C were previously considered to describe the phenotypic responses, see Section 2.2.1.). Selected data were normalized as in Section 2.2.1. Transformed data were superimposed on the General, SF and the RSM model. The validity domain of these models was tested as described for HO and WT models.

### 2.3 Data extraction and statistical analysis

Data taken from published scatter graphs (e.g. Canvin, 1965; Fernández-Moyá et al., 2002; Baux et al., 2013) were extracted by using the XYScan program (2010). Normality and variances homogeneity of each established model was analyzed (alpha = 0.05). Fits were made using R-Studio, Sigmaplot 12.0 (Sigmaplot, 2016) (SPSS Inc., Chicago, Illinois) and Infostat (Di Rienzo et al., 2008) software.

A detailed analysis in each temperature interval was performed on DataSet A to discard artifacts in the analysis due to the data imbalance (Chaplin-Kramer et al., 2011). First, the mean value of fatty acid percentage in every temperature interval was determined for each species. Second, the average of the percentage of each fatty acid from the whole data set was determined in each temperature interval. Finally, the mean value of each species and the general mean value of each temperature interval were compared (Student’s t-test) to determine the degree of contribution of each species to the general response. Parallelism analysis (Kobayashi and Salam, 2000) was performed to compare the slopes and ordinates to the origin of the different lineal models.

## 3 Results

### 3.1 General Model of fatty acid response to temperature

**Figure 1:**
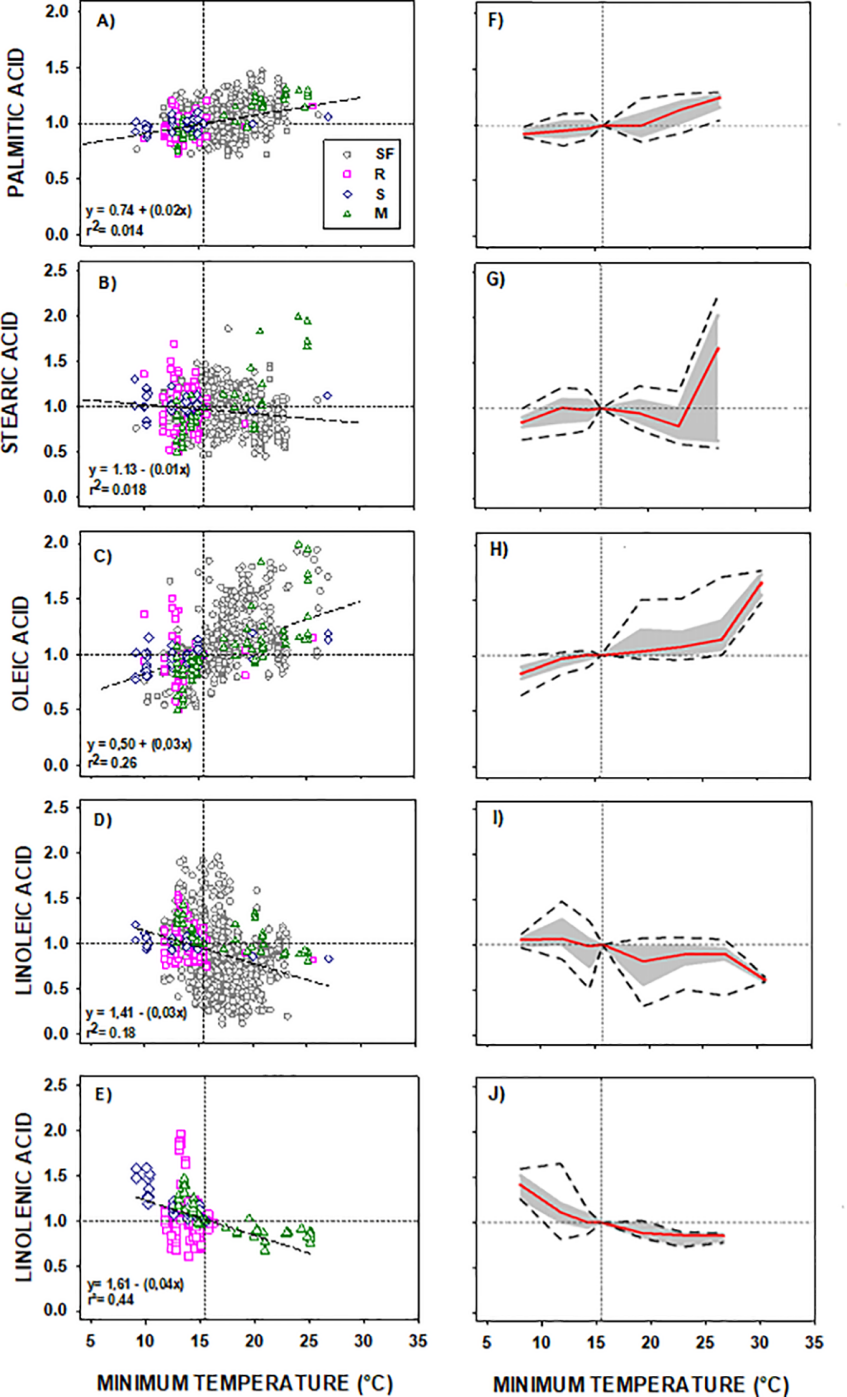
**(A-J)** General Model of the phenotypic response of the seed oil fatty acid to minimum temperature of four oilseed crop species. Left panels: data distribution of Sunflower (
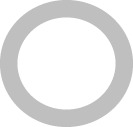
 SF), Rapeseed (
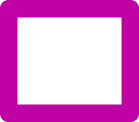
 R), Soybean (
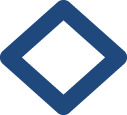
 S) and Maize (
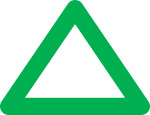
 M). Scatter line indicated significant fittings (p<0.001). Right panels: phenotypic response of the fatty acid to minimum temperature of the four crop species. In both charts: horizontal line represents the “non-response threshold” of the plotted data; vertical dotted line indicates the reference value of minimum temperature (15.5 °C) for normalization.

Based on the collected information, common responses of the fatty acids to temperature were established for the group of species and genotypes (see Section 2.1). Consistent phenotypic responses were observed across all fatty acids in genotypes of the four species included in DataSet A (comprising several genotypes from four species sunflower, rapeseed, soybean, and maize, see Section 2.1) (**Figure 1**). The left panels of **Figure 1** show the distribution of points by species for phenotypic responses across a common temperature range (8.7 – 27.0 °C). The right panels illustrate the reaction norms for each fatty acid, delineating the shape of their phenotypic response, depicting the direction, the magnitude, and the extent of the phenotypic change. Notably, saturated fatty acids exhibited lower phenotypic responses compared to unsaturated ones as evidenced for their lower slopes (<0.02 *vs*. >0.03 for saturated and unsaturated acids, respectively, **Figure 1**, left panels) and their lower phenotypic plasticity (especially below 21°C, **Figure 1**, right panels).

Palmitic and stearic acids showed a minimal trend to increase or decrease with decreasing Tmin, respectively (**Figures 1A, B**). Saturated fatty acids presented almost no plasticity below 17°C (**Figures 1F, G**). Its direction and magnitude of the response increased at higher temperature, particularly for the stearic acid at temperatures above 21°C (**Figures 1B, G**).

Oleic and linoleic acids exhibited high phenotypic responses. The trend line slopes, the magnitude and the extent of the phenotypic change of the reaction norms were substantial at temperatures above 15°C. Specifically, oleic acid demonstrated a clear increase with rising Tmin (**Figure 1C**) and a high extent of the phenotypic change (**Figure 1H**) at temperatures exceeding 20°C. Linoleic and linolenic acids, on the other hand, decreased with increasing Tmin (**Figures 1D, E**). The reaction norm of linoleic acid displayed high phenotypic plasticity across the entire temperature range (**Figures 1D–I**). A similar phenotypic response was observed for linolenic acid (present in rapeseed, soybean, and maize seed oils) except that the magnitude of its reaction norm increased at temperatures below 15°C (**Figures 1E–J**).

The contribution of each species’ data to DataSet A (left panels of **Figure 1**) did not significantly influence (p < 0.01) the trends between fatty acids and minimum temperature, despite sunflower being the largest contributor to the dataset (50.8%). Although in some intervals data were not registered for all species (e.g., maize data missing in the 8 – 10°C interval), this did not affect the general trend (p < 0.05). Instances where a species was not registered in one interval were compensated by the presence of data of the same species in adjacent intervals (e.g., maize data absent at 8 – 10°C but present from 11 to 13°C). Rapeseed and soybean data covered temperature ranges below 20°C, while sunflower and maize ranged almost the entire temperature spectrum explored.

### 3.2 Several responses of fatty acids to temperature varied between sunflower and combined data of rapeseed, soybean and maize

DataSet A was partitioned into i) sunflower data (SF-Model) and ii) combined rapeseed, soybean, and maize data (RSM-Model) due to sunflower heightened Tmin sensitivity for certain fatty acids (e.g., oleic and linoleic acids) compared to other species (see **Figure 1** point distribution).

**Figure 2:**
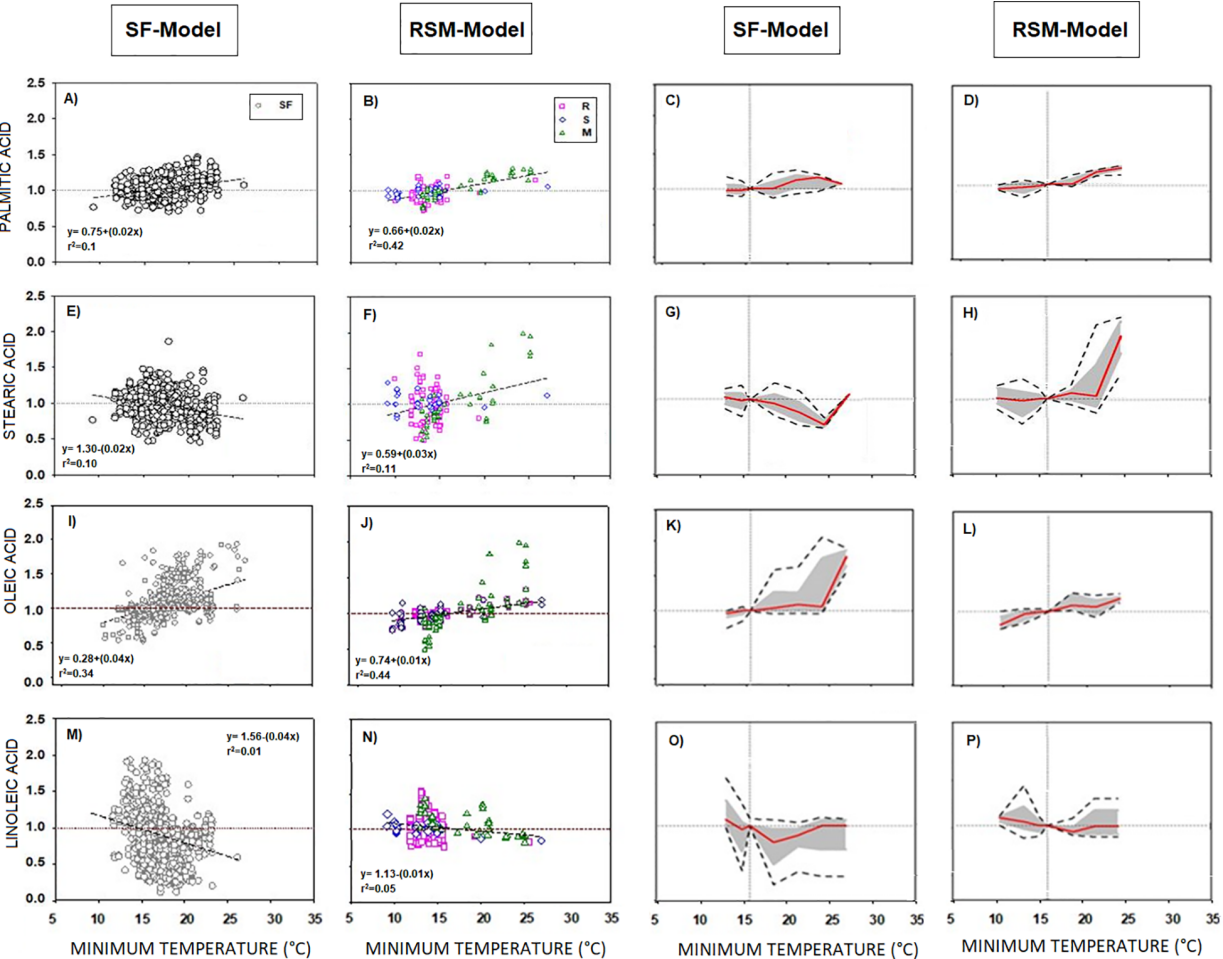
**(A-P)** SF Model (Sunflower, 
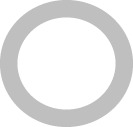
 SF) and RSM Model (Rapeseed, 
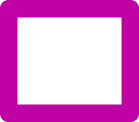
 R + Soybean, 
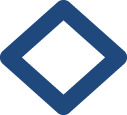
 S + Maize, 
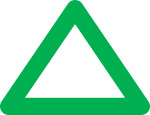
 M) of the phenotypic response of the seed oil fatty acid to minimum temperature. Columns 1 and 2 correspond to data distribution of SF-Model and RSM-Model. Scatter line indicated significant relationship between them (p-value <0.001). Columns 3 and 4 correspond to the reaction norms of the fatty acids to minimum temperature of SF and RSM. Horizontal line represents the “non-response threshold” of the plotted data; vertical dotted line indicates the reference value of minimum temperature (15.5 °C) on the independent axis.

Response patterns to minimum temperature were similar across all fatty acids in both SF-Model and RSM-Model. However, RSM-Model differs from SF-Model in some cases. Stearic acid displayed a negative trend in SF-Model and positive trends in RSM-Model (**Figures 2E, F**). Reaction norms in RSM-Model generally showed greater extent of the phenotypic change compared to SF-Model (**Figures 2C, D, G, H**).

Distinct differences between SF and RSM-Models were evident in unsaturated fatty acids like oleic and linoleic acids. Both fatty acids (**Figures 2I, J, M, N**) stood out for their higher temperature responses compared to saturated fatty acids (**Figures 2A, B, E, F**). In SF-Model, oleic acid displayed a steeper slope than in RSM-Model. The magnitude of its reaction norm remained low below 23°C but increased thereafter (**Figure 2K**), while the extent of the phenotypic change was high across almost the whole temperature range. Conversely, in RSM-Model, both the magnitude and extent of phenotypic change were low (**Figure 2L**). Linoleic acid response to Tmin similarly exhibited a steeper slope in SF-Model than in RSM-Model (**Figures 2M, N**), with similar direction and magnitude of phenotypic plasticity (**Figures 2O, P**), but greater extent of phenotypic change in SF-Model.

### 3.3 The responses of fatty acids to temperature in plants grown under field and controlled conditions are similar, except for oleic acid in traditional sunflower

Comparing field and controlled conditions data (DataSet A), slopes and intercepts of fatty acid responses to Tmin showed no significant differences (**Table 4**), except for oleic acid. In this sense, oleic acid under controlled conditions showed a steeper slope compared to field conditions when analyzing the species together. Considering sunflower on one side and RSM on the other, significant differences were observed in sunflower’s oleic acid response to temperature between field and controlled conditions, but not in the other group of species (**Table 5**). Under controlled conditions, a steeper slope and a lower intercept were observed compared to field conditions in sunflower genotypes.

**Table 4:** Comparison of ordinates to the origin and slopes of the fittings of every seed oil fatty acid *vs*. temperature of plants grown under field and control conditions during oil synthesis (DataSet A).

	Fatty acid	Tmin	*p-value*	FA: Tmin	*p-value*
Palmitic acid	Field	0.71	0.16	0.017	p= 0.77
	Control conditions	0.79		0.017	
Stearic acid	Field	0.98	0.37	0.003	0.89
	Control conditions	1.11		0.002	
Oleic acid	Field	0.58	0.08	0.027	0.02*
	Control conditions	0.44		0.038	
Linoleic acid	Field	1.47	0.19	-0.03	0.16
	Control conditions	1.64		-0.04	
Linolenic acid	Field	1.47	0.17	-0.02	0.64
	Control conditions	1.21		-0.02	

**Table 5:** Comparison of ordinates to the origin and slopes of the fittings of seed oil oleic fatty acid *vs*. temperature for sunflower data *vs*. combined data of rapeseed, soybean and maize grown under field and control conditions during oil synthesis.

	Fatty acid	Tmin	*p-value*	FA: Tmin	*p-value*
SF	Field	0.52	<0.0001	0.03	<0.0001
	Control conditions	0.12		0.06	
RSM	Field	0.79	0.62	0.01	0.53
	Control conditions	0.88		0.01	

### 3.4 The high oleic trait only influences the response to temperature of the oleic and linoleic acids

To assess the impact of the high oleic trait on the phenotypic responses of oil fatty acids to minimum temperature, DataSet A was split into traditional (WT) and high oleic (HO) genotypes, regardless of the species.

**Figure 3:**
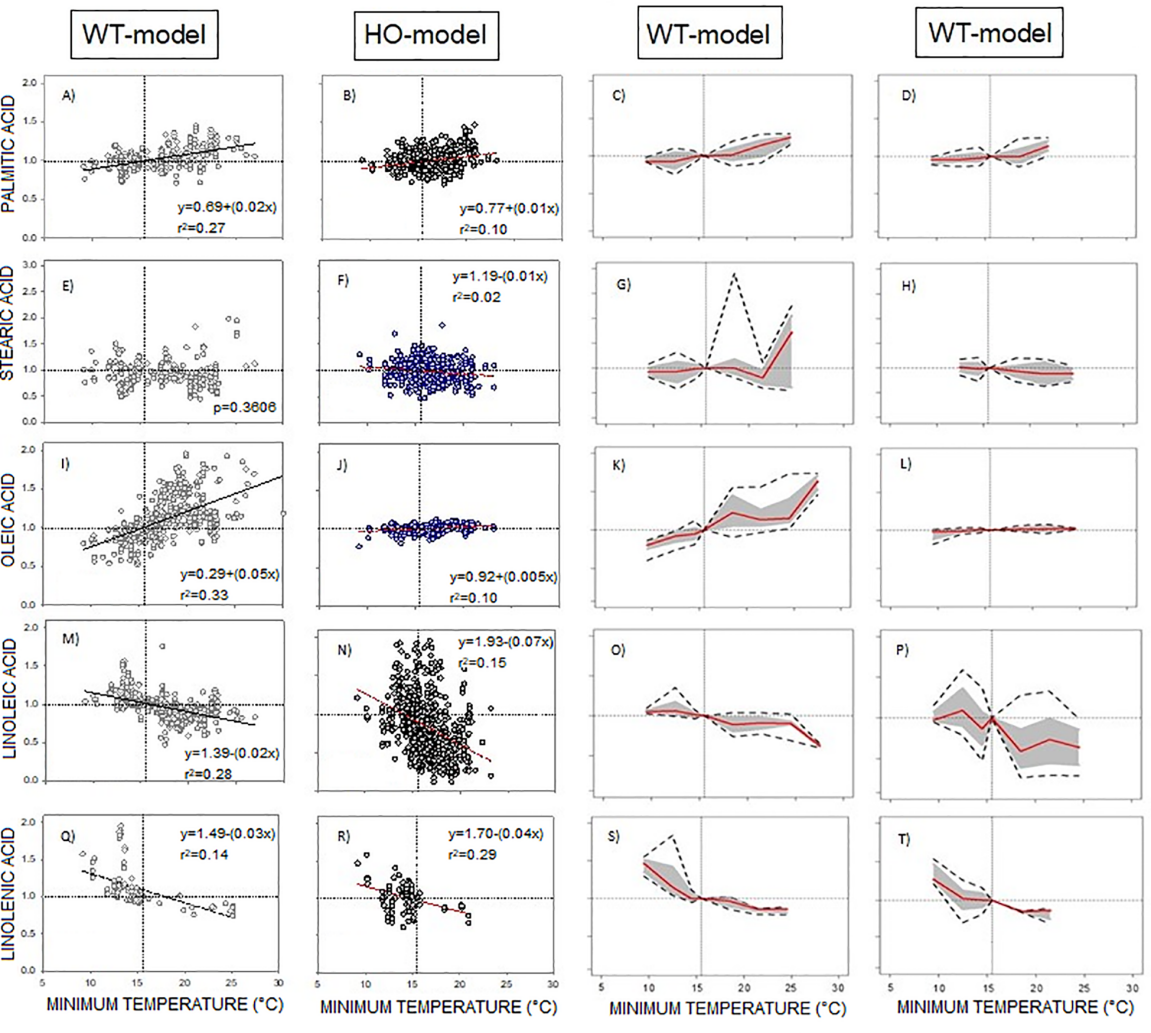
**(A-T)** WT-Model and HO-Model of the phenotypic response of the seed oil fatty acid to minimum temperature. Columns 1 and 2 correspond to data distribution of WT-Model (
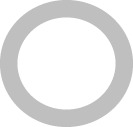
) and HO-Model (
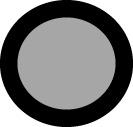
) respectively. Scatter line indicated significant relationship between them (p-value <0.001). Columns 3 and 4 correspond to the reaction norms of the fatty acids to Tmin of WT-Model and HO-Model. Horizontal line represents the “non-response threshold” of the plotted data; vertical dotted line indicates the reference value of minimum temperature (15.5 °C) on the independent axis.

The presence of the HO trait minimally modified the response of saturated fatty acids and linolenic acid to temperature (**Figure 3**). Also, the reaction norms of these fatty acids exhibited similar response patterns between WT-Model and HO-Model (**Figure 3**).

The responses of oleic and linoleic acids to temperature were significantly modified by the HO trait (**Figures 3J–N**). In HO-Model, the slope of the oleic acid response to Tmin was statistically non-significant (p = 0.36). Accordingly, the point distribution was small (**Figure 3J**) and the reaction norm was null (**Figure 3L**). In contrast, WT-Model showed a steep oleic acid to Tmin response slope (0.05). Its reaction norm magnitude was high above 16°C (**Figure 3K**) but decreased at lower temperatures. Linoleic acid response to temperature was also markedly influenced by the HO trait, displaying a steeper slope in HO than in WT (**Figures 3M, N**). Reaction norms for both WT and HO exhibited similar direction, but HO displayed higher magnitude (**Figure 3P**).

Further analysis of the linoleic acid phenotypic response to Tmin in HO-Model was performed. The DataSet was split in sunflower WT (SF-WT), sunflower HO (SF-HO), RSM-WT and RSM-HO. The direction of the reaction norms was similar between SF-WT and RSM-WT and between SF-HO and RSM-HO. The magnitude of the phenotypic change was however higher in SF-HO than in RSM-HO (**Supplementary Figure 3**).

### 3.5 Impact of traits other than HO trait on fatty acid response to temperature

The effect of traits other than HO trait (DataSet B, comprising genotypes carrying at least one trait affecting fatty acid composition, different from the HO trait; e.g., HS: high stearic, HP: high palmitic, HL: high linoleic, and LL: low linolenic; see section 2.1) on phenotypic responses of fatty acids to temperature was examined using the previously established WT and HO models (Section 3.4) as analytical tools. In most of tested genotypes these traits were combined with the HO trait.

**Figure 4:**
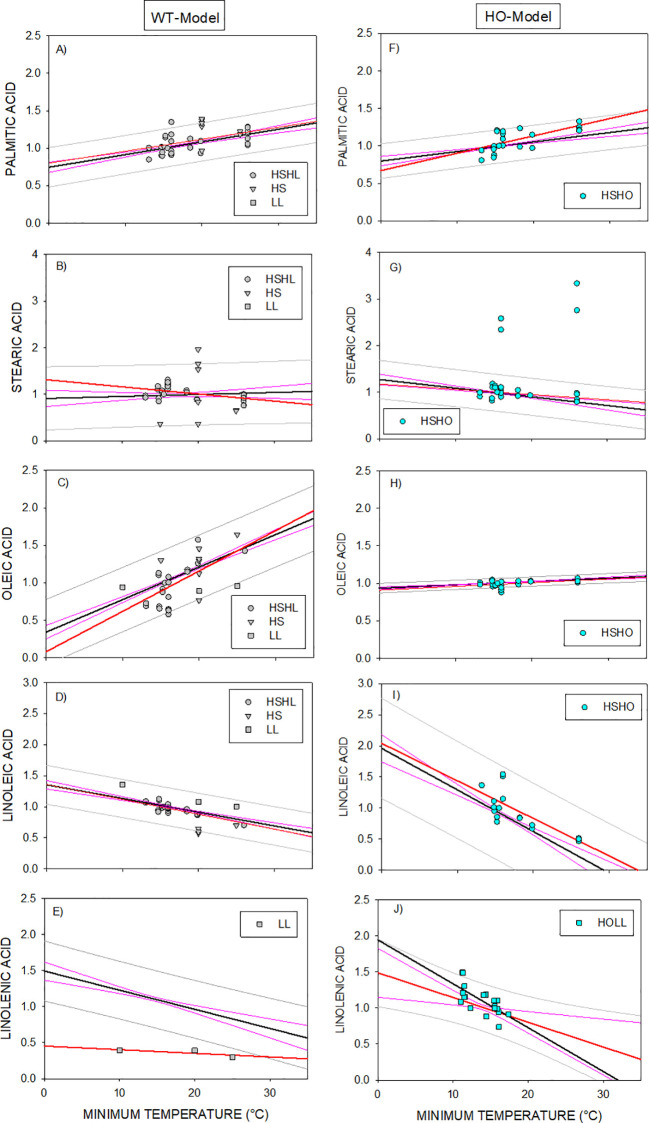
**(A-J)** Superimposition of data of genotypes carrying the high stearic (
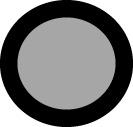
 HS), high linoleic (
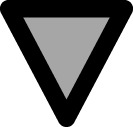
 HL), low linolenic (
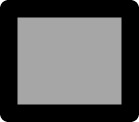
 LL), high stearic/high oleic (
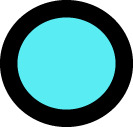
 HSHO) or high oleic/low linolenic (
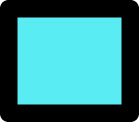
 HOLL) traits to the overall phenotypic responses in WT-Model (left panels) and HO-Model (right panels). Grey and pink lines correspond to the prediction and confidence intervals of the WT and HO models. Bold black lines correspond to the regression lines of the WT or HO models. Data were superimposed on HO-Model or on WT-Model depending on it included or nor the HO trait, respectively. Bold/red lines corresponded to the regression lines fitted to the set of independent genotypes.

Data from DataSet B were overlapped to the established phenotypic responses of WT or HO Models according to if the genotype carried or not the HO trait, respectively. Superimposed data were in most of cases (93.7% of the whole DataSet B) within the prediction intervals of the respective model (**Figure 4**). For every fatty acid, the fittings to the independent data from DataSet B were also within the prediction intervals of responses of WT or HO models (**Figure 4**). Slopes and ordinates of fittings to data from DataSet B were similar to those from responses established for the respective WT or HO Model (**Supplementary Table 1**).

The analyzed data similarly behaved independently of the considered trait (which was different from HO trait). Further, it was still maintained when the genotype also included the HO trait. Moreover, the response of the fatty acids synthetized upstream or downstream of the mutated trait was not altered by its presence(**Figure 4**). Only a few data points were located outside the prediction intervals of the previously established models (4.6% and 1.4% of data of DataSet B were outside the prediction intervals of the WT-Model and the HO-Model, respectively). In most cases, this occurred when the phenotypic response of a fatty acid concurred with the coding trait (i.e. HS trait in **Figure 4B** and LL trait in **Figure 4E**). A similar behavior was observed in genotypes that presented a trait combined with HO trait. For instance, HSHO data were located outside the prediction intervals of the stearic acid HO-Model (**Figure 4G**).

### 3.6 Testing the validity of established response models against data from other independent species

The validity domain of the responses of fatty acids to temperature included in the models established here above from DataSet A was tested against the responses to temperature of independent genotypes included in DataSet C. This database included genotypes of Flax, Chia, Safflower, Camellia and Olive (oil fatty acids from seed and mesocarp) grown under a wide range of environmental conditions. The tested models were established by using genotypes from sunflower, rapeseed, soybean and maize (sections 3.1, 3.2 and 3.3). Some genotypes from Safflower, Camellia and Olive (DataSet C) were not considered in the analysis as the ranges of temperatures explored by their data were < 2°C (see M&M Section 2.2.1).

**Figure 5:**
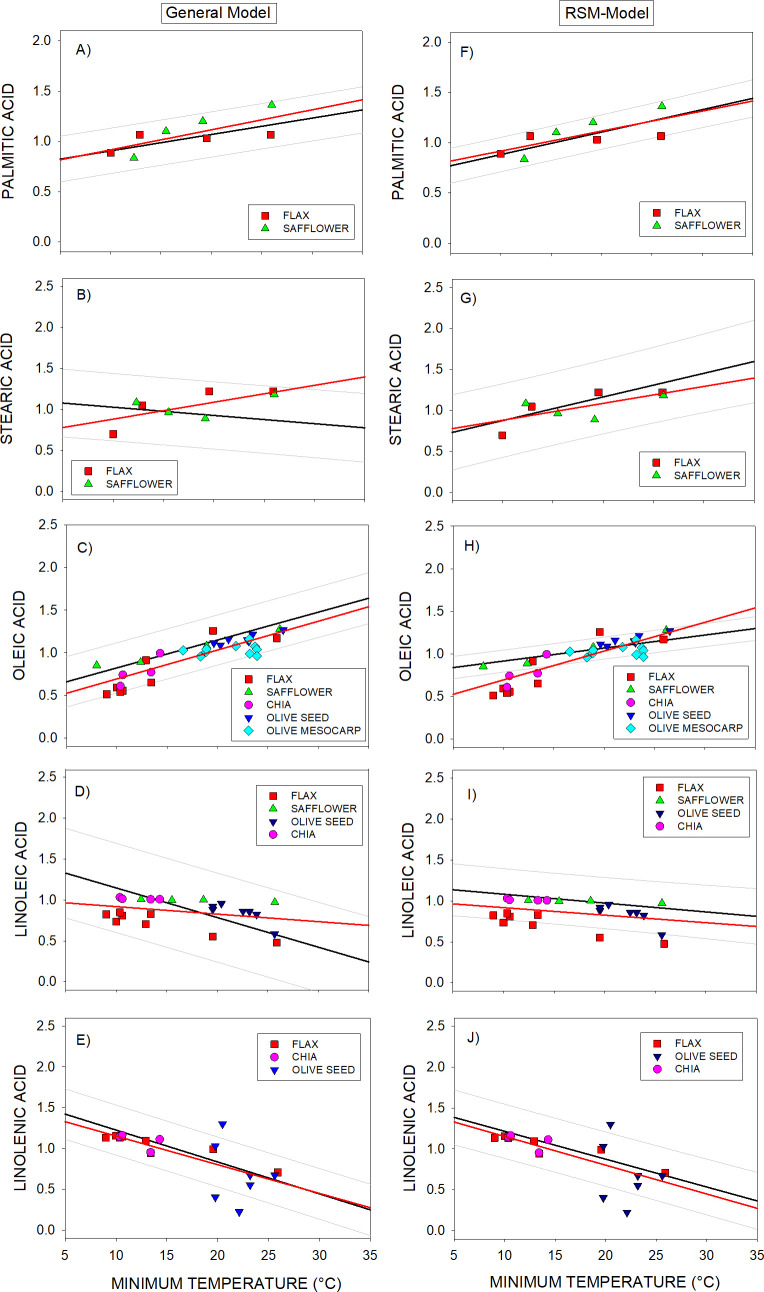
**(A-J)** Assessment of responses of the General-Model (left panels) and RSM-Model (right panels) using independent species (DataSet C). Bold/black lines corresponded to the regression lines of the General or RSM models. Symbols correspond to: 
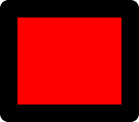
 Flax, 
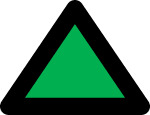
 Safflower, 
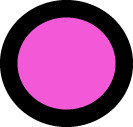
 Chia, 
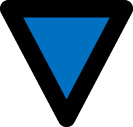
 Olive seed, 
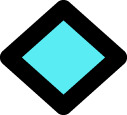
 Olive mesocarp. Grey lines correspond to the prediction intervals of the General and RSM models. Data were superimposed on General-Model and on RSM-Model. Bold/red lines corresponded to the regression lines of set of independent species.

In 97% of cases the independent data of the whole DataSet C were within the prediction intervals of the General Model (**Figure 5**). The only exception was three data points of the linolenic acid of olive seed (**Figure 5E**). Also, the responses of fatty acids to temperature were in accordance with those from the General Model (**Figures 5B–D**). However, the responses of the stearic and linoleic acid to temperature were better described by the RSM-Model (**Figures 5F–I**). Though, the 15% of points corresponding to oleic, linoleic and linolenic acids altogether were outside the prediction intervals of the RSM-model.

The response to temperature of the independent species individually analyzed resulted well aligned with the General Model. The only exception was the stearic acid of flax that better fitted to the WT or RSM Model (considering only WT genotypes) than to the General Model (**Figures 5B–G**) (**Supplementary Figures 4A, B**; **Supplementary Table 2**).

## 4 Discussion

Common patterns of fatty acid responses to temperature were identified across a broad spectrum of genotypes from nine species using a previously unavailable database (**Tables 1**–**3**; Repository). This database exhibits significant genotypic variation in various traits: i) plant morphology and architecture (e.g., from herbs to trees, branched and unbranched species, genotypes with determinate and indeterminate growth, planophiles, extremophiles, and erectophiles), ii) fruit types (achenes, pods, siliques, fleshy, gourds, cypselae), iii) plant physiology (e.g., autotrophic and heterotrophic fruits, C3 and C4 photosynthetic metabolisms), and iv) centers of origin (e.g., temperate *vs*. tropical environments). Additionally, the database includes genotypes carrying single and/or double mutations modifying their fatty acid composition (e.g., high oleic, high oleic/low linolenic mutations). Data were gathered from plants grown under diverse environmental conditions (e.g., in field settings across different locations and sowing dates, in greenhouses, or growth chambers). Statistical analyses confirmed that the database was balanced. Although sunflower was the most represented species, the established models were not spurious due to an unequal distribution of species in the database.

The meta-analysis conducted in this study investigated the response of fatty acids to temperature, despite variations of more than tenfold in the percentages of some fatty acids among the studied genotypes and species (e.g., 7-93% for oleic acid, 0.7-92% for linoleic acid). Meta-analytic approaches have previously been employed successfully to study the variability and environmental responses of other traits (Poorter et al., 2010; Challinor et al., 2014; Van and McHale, 2017; de Borja Reis et al., 2021; Secchi et al., 2023; Ngidi et al., 2024). This work extends its application to the response of fatty acids to temperature across a wide range of genotypes from different species, cultivated under varying temperature conditions during the oil synthesis stage, essential to produce oils with better quality for human consumption. Moreover, the methodology used in this work to establish common responses of seed oil fatty acids to temperature could be applied to analyze other traits. For instance, it could be applied to study the membrane fatty acids changes in leaves with temperature variations (e.g. Falcone et al., 2004), and, consequently, determine the acclimation temperature for a set of species and genotypes.

Saturated fatty acids consistently exhibited a lower response to temperature compared to unsaturated fatty acids across all established models. This work generalizes the differences in responses between genotypes of multiple species, although the analyzed works have carried out empirical research on a reduced number of genotypes in a few species (e.g., Baux et al., 2008, 2013; Agosti, 2011; Zuil et al., 2012; Schulte et al., 2013; Regitano Neto et al., 2016; Angeloni et al., 2017). The differential responsiveness of saturated and unsaturated fatty acids to temperature could be attributed to variations in the thermal sensitivity of the enzymatic complexes involved in their synthesis. FAS II (responsible for the elongation of palmitic to stearic acids) and SAD (responsible for the desaturation of stearic to oleic acids) exhibit slight temperature lability (Martínez-Force et al., 1998). FAD2 (responsible for the desaturation of oleic to linoleic acids) and FAD3 (responsible for the desaturation of linoleic to linolenic acids) are significantly sensitive to temperature variations (Martinez-Rivas et al., 2001; Cahoon and Schmid, 2008). This was observed by other authors in the species analyzed here [e.g., soybean (Zhao et al., 2019), maize (Narayanan et al., 2020), olive (D’Angeli and Altamura, 2016), flax (Pushkova et al., 2024)] and in other species not considered in this work due to their low oil content [e.g., banana (Cheng et al., 2022), cucumber (Dong et al., 2016)].

Different unsaturated fatty acids demonstrated varying degrees of sensitivity to temperature. The response of oleic acid was more pronounced compared to that of linoleic and linolenic acids. This discrepancy may be attributed to the higher thermal sensitivity of the FAD2 complex, which decreases its activity with increasing temperatures (Garcés et al., 1992; Martínez-Rivas et al., 2000, 2001). Conversely, the FAD3 complex, responsible for the desaturation of linoleic to linolenic acid, exhibits considerably lower sensitivity to temperature (Vuorinen et al., 2014). Furthermore, the response of oleic acid to temperature varied significantly among species, indicating genetic variability in the sensitivity of oleic acid to temperature, even within wild-type genotypes. Overall, the findings of this study support the idea that not only have genes involved in fatty acid synthesis pathways been conserved through evolution and genetic improvement (Celik Altunoglu et al., 2018), but also that the response of different fatty acids to temperature is preserved.

Considering the current context of climate change, where increases in minimum temperature are observed, a decrease in the degree of oil unsaturation in seeds of diverse species has been evidenced. While this may be considered positive from a consumption standpoint, it could have a negative impact on the seed when planted. In this sense, the temperature of the zone where the seed is produced determines its fatty acid composition, its saturation index, and therefore the rate at which it will germinate, depending on whether it is sown in a colder or warmer region (Izquierdo et al., 2017).

Oleic acid in HO genotypes across different species did not exhibit a response to temperature, as evidenced by comparisons between HO and WT models. Previous studies have reported that the HO trait decreases the temperature responsiveness of oleic acid (e.g., Baux et al., 2008; Izquierdo and Aguirrezábal, 2008; Alberio et al., 2018). However, conflicting results have been observed in some genotypes of soybean and sunflower (e.g., Zuil et al., 2012; Angeloni et al., 2017). The results presented here generalize that a positive temperature response of oleic acid is attributable only to WT genotypes, whereas it is null in those carrying the HO trait. Therefore, the findings also indicate that breeding efforts have successfully improved oil quality. Including the HO trait in genotypes from various species has increased the levels of oleic acid (e.g., Soldatov, 1976; Leon et al., 2013), while also decreasing its temperature responsiveness, nearly eliminating it. Thus, HO genotypes can maintain stable oil quality almost independently of the growth environment.

Other traits apart from HO did not alter the response of fatty acids synthesized upstream or downstream of the mutation in the fatty acid synthesis pathway. Both in genotypes carrying single (HS or HL) or double mutations (HSHL or HOLL), the responses of all fatty acids to temperature fell within the predicted ranges of those responses included in the appropriate models (HO or WT). The few responses outside these predicted ranges (6% of cases) were detected when the phenotypic response of a fatty acid coincided with the coding trait. The non-effect of these traits different than HO on the temperature responses of other fatty acids has not been previously reported. These results extend the validity of the HO or WT models to genotypes of sunflower and rape carrying mutations other than HO, as well as when combined with the HO trait. Thus, they demonstrate that the models established in this study could be utilized as tools to assess the impact of including mutations in the fatty acid synthesis pathway (e.g., for predicting the temperature response of fatty acids for “in silico” genotypes carrying single or double mutations).

The establishment of distinct models from subsequent dissections of the dataset facilitated the identification of previously unknown behaviors in the responses of fatty acids to temperature. For instance, in HO genotypes, the response of linoleic acid to temperature (synthesized from oleic acid) was enhanced. This finding was corroborated by the responses of linoleic acid in SF-HO and RSM-HO models. However, the HO trait did not influence the responses of fatty acids synthesized downstream (linoleic and linolenic acids) or upstream (SF and RSM) of oleic acid. Interestingly, the effect of the HO mutation on the temperature response of linoleic acid observed in HO sunflower genotypes was not observed when the HO trait was combined with other mutations in the fatty acid pathway. Thus, it is suggested that the increased temperature response of linoleic acid in SF-HO could be nullified when the HO trait is combined with other mutations. Empirical research to test this model-based hypothesis could be valuable for verifying this suggestion.

Plants grown under field conditions and those under controlled conditions exhibited similar temperature responses for all fatty acids across genotypes of different species, except for the response of oleic acid in WT genotypes of sunflower. This novel finding is significant, as it has been debated whether results obtained under controlled conditions can be extrapolated to predict plant responses in the field (i.e., Poorter et al., 2012, 2016). However, the results of this study support those investigations into the trait “response of oil fatty acids to temperature” can be conducted indistinctly under controlled conditions, in the field, or by merging data obtained under both conditions. Overall, this new knowledge could aid in designing more cost-effective and less time-consuming experiments.

The General Model can be used to determine the response of different fatty acids in all species and genotypes considered in this study. Since all genotypes of the species tested for validation were WT, the response of certain fatty acids better fit with the WT model or RSM model (WT) than with the General or RSM model (which includes HO genotypes). In olive, different trends were observed in the response of oleic acid to temperature when considering the mesocarp or the seed in the tested models. However, the data for both olive seeds and mesocarps fell within the prediction intervals of the relationships included in the four models. Moreover, these models could help identify favorable environments for producing seeds that germinate better under low-temperature conditions. Indeed, the germination of various species, including canola, sunflower, wheat, soybean, safflower, flax, cotton, quinoa, rice, among others, is related to the iodine index (which measures oil unsaturation), with linoleic acid particularly important for sunflower (Izquierdo et al., 2017). These favorable environments could be identified for current and future climate scenarios by combining the models presented in this study with the methods applied by Angeloni et al. (2017).

The meta-analytic method employed in this study revealed some limitations when the response of fatty acids was low [e.g., nearly HO isolines of the same species (Alberio et al., 2018)] or when the range of temperature variation during the experiment was narrow [e.g., <2°C (Rondanini et al., 2014; Zemour et al., 2021; Abou Chehade et al., 2022)]. These limitations arose because the temperature range explored here was 17°C (8.7 - 27.0°C), and the intervals used to establish the models were fixed at 2°C (tolerance error = 1.5°C). Thus, the validity of the established models could not be tested for some genotypes of olive, safflower, and camellia, as their data covered a temperature range of <2°C. Reducing the temperature intervals from 2°C to 1°C could increase the errors in the response slopes of different fatty acids to temperature. Moreover, decreasing the total range of explored temperatures to accommodate the excluded genotypes would reduce the number of species considered in the established models.

Sunflower has traditionally been considered as a “model” oilseed species for studying the environmental effects on fatty acid composition (Aguirrezábal et al., 2015). However, sunflower WT genotypes exhibited i) a more pronounced response of fatty acids to temperature than genotypes of other studied species and ii) a different temperature response of oleic acid between plants grown under controlled conditions and those in the field. Additionally, an effect of the HO trait on the response of linoleic acid to temperature was detected exclusively in sunflower. Therefore, it would be suggested that species other than sunflower should be considered as models for studying the response of fatty acids to environmental conditions. Rapeseed would be potentially a candidate because i) its fatty acid response to temperature was similar to that of genotypes of other species studied here, ii) its oil contains measurable linolenic acid (unlike sunflower), iii) it is globally cultivated, and iv) its genetics are closer to Arabidopsis thaliana (the model species in plant science) than sunflower (Delourme et al., 2006). However, this suggestion would need to be confirmed with further investigation of the response of rapeseed oil fatty acids to minimum temperatures above 16°C, whose process could be accelerated by using the methodology and tools developed here. A current challenge in plant science is linking genotype to phenotype (Barba-Espin and Acosta-Motos, 2022). However, phenotyping is laborious and time-consuming (Hall and Richards, 2013; Tardieu and Parent, 2017). By employing a meta-phenomic approach (e.g., Poorter et al., 2022), this study provides methods, novel insights, and tools to facilitate more efficient, rapid, and cost-effective phenotyping of fatty acid responses to temperature (Fiorani and Schurr, 2013; Tardieu and Parent, 2017; Peirone et al., 2018; Pieruschka and Schurr, 2019). The results presented here could aid in assessing *a priori* the potential response of fatty acids to temperature in species and genotypes not previously studied, using a small number of points that could be obtained from experiments carried out under controlled conditions, as results presented previously suggested. This could be particularly significant for “orphan” crops, which have niche markets in local economies but limited research. These crops have been underexplored but may present new opportunities in the context of climate change (Mabhaudhi et al., 2019).

## 5 Conclusions

We presented the effectiveness of several phenotypic models in summarizing the temperature responses of seed oil fatty acids across a range of crop species using meta-phenomics. The established models, particularly the General Model, that includes data from seed oil fatty acids of sunflower, rapeseed, soybean and maize, exhibited robustness in predicting the phenotypic responses of palmitic, stearic, oleic, linoleic, and linolenic acids in oils derived from a wide array of globally cultivated species and orphan crops. These models possess broad applicability, enabling extrapolation to predict fatty acid responses to temperature in genetically and morphologically distinct species, as well as in genotypes harboring mutations within the fatty acid biosynthetic pathway, such as sunflower or rapeseed with minimal effort devoted to experimental validation.

The standardized models of fatty acid response to temperature delineated in this research are poised to inform future endeavors aimed at improving oil quality across different species and genotypes. Additionally, they offer a framework for designing streamlined, efficient experiments that require less time and resources.

This work is the first attempt to the establishment of a general response model of the seed fatty acid to temperature. Additional research accounting for the response of fatty acid to temperature in species less or not considered in this work will be needed to increase the robustness of the analysis conducted here, allowing researchers and breeders alike to help farmers predict the outcomes they see when growing different oilseed crops.

## Data Availability

The datasets presented in this study can be found in online repositories. The names of the repository/repositories and accession number(s) can be found below: https://figshare.com/, https://doi.org/10.6084/m9.figshare.24994631.v1.

## References

[B1] Abou Chehade L. Angelini L. G. Tavarini S. (2022). Genotype and seasonal variation affect yield and oil quality of safflower (Carthamus tinctorius L.) under Mediterranean conditions. Agron. 12, 122. doi: 10.3390/agronomy12010122

[B2] Agosti M. B. (2011). Fertilización nitrógeno-azufrada y variabilidad genotípica en el rendimiento y la calidad de grano en colza-canola (Brassica napus L.). Buenos Aires: Universidad de Buenos Aires.

[B3] Aguirrezábal L. Martre P. Pereyra-Irujo G. Echarte M. M. Izquierdo N. (2015). “Improving grain quality: ecophysiological and modeling tools to develop management and breeding strategies,” in Crop physiology, 423–465.

[B4] Aguirrezábal L. Martre P. Pereyra-Irujo P. Izquierdo N. Allard V. (2009). “Management and Breeding Strategies for the Improvement of Grain and Oil Quality,” in Crop physiology: applications for genetic improvement and agronomy (San Diego, CA, USA: Academic Press), 387–421.

[B5] Ahmad M. Waraich E. A. Skalicky M. Hussain S. Zulfiqar U. Anjum M. Z.. (2021). Adaptation strategies to improve the resistance of oilseed crops to heat stress under a changing climate: An overview. Front. Plant Sci. 12, 767150. doi: 10.3389/fpls.2021.767150 34975951 PMC8714756

[B6] Alberio C. (2017). Bases ecofisiológicas para la obtención de aceites de canola y girasol con calidad diferenciada. Mar del Plata: Universidad Nacional de Mar del Plata.

[B7] Alberio C. Aguirrezábal L. A. Izquierdo N. G. Reid R. Zuil S. Zambelli A. (2018). Effect of genetic background on the stability of sunflower fatty acid composition in different high oleic mutations. J. Sci. Food Agric. 98, 4074–4084. doi: 10.1002/jsfa.2018.98.issue-11 29388684

[B8] Alberio C. Izquierdo N. G. Galella T. Zuil S. Reid R. Zambelli A.. (2016). A new sunflower high oleic mutation confers stable oil grain fatty acid composition across environments. Eur. J. Agron. 73, 25–33. doi: 10.1016/j.eja.2015.10.003

[B9] Angeloni P. Echarte M. M. Irujo G. P. Izquierdo N. Aguirrezábal L. (2017). Fatty acid composition of high oleic sunflower hybrids in a changing environment. F. Crops Res. 202, 146–157. doi: 10.1016/j.fcr.2016.04.005

[B10] Arnold P. A. Kruuk L. E. Nicotra A. B. (2019). How to analyse plant phenotypic plasticity in response to a changing climate. New Phytol. 222, 1235–1241. doi: 10.1111/nph.2019.222.issue-3 30632169

[B11] Aslam M. N. Nelson M. N. Kailis S. G. Bayliss K. L. Speijers J. Cowling W. A. (2009). Canola oil increases in polyunsaturated fatty acids and decreases in oleic acid in drought-stressed Mediterranean-type environments. Plant Breed. 128, 348–355. doi: 10.1111/j.1439-0523.2008.01577.x

[B12] Ayerza R. (1995). Oil content and fatty acid composition of chia (Salvia hispanica L.) from five northwestern locations in Argentina. J. Am. O. Chem. Soc 72, 1079–1081.

[B13] Barba-Espin G. Acosta-Motos J. R. (2022). Crop genetic resources: an overview. Agron. 12, 340. doi: 10.3390/agronomy12020340

[B14] Baux A. Colbach N. Allirand J. M. Jullien A. Ney B. Pellet D. (2013). Insights into temperature effects on the fatty acid composition of oilseed rape varieties. Eur. J. Agron. 49, 12–19. doi: 10.1016/j.eja.2013.03.001

[B15] Baux A. Hebeisen T. Pellet D. (2008). Effects of minimal temperatures on low-linolenic rapeseed oil fatty-acid composition. Eur. J. Agron. 29, 102–107. doi: 10.1016/j.eja.2008.04.005

[B16] Beló A. Zheng P. Luck S. Shen B. Meyer D. J. Li B.. (2008). Whole genome scan detects an allelic variant of fad2 associated with increased oleic acid levels in maize. Mol. Gen. Genom. 279, 1–10. doi: 10.1007/s00438-007-0289-y 17934760

[B17] Bradshaw A. D. (1965). Evolutionary significance of phenotypic plasticity in plants. Adv. Gen. 13, 115–155. doi: 10.1016/S0065-2660(08)60048-6

[B18] Brandán J. P. Izquierdo N. Acreche M. M. (2022). Oil and protein concentration and fatty acid composition of chia (Salvia hispanica L.) as affected by environmental conditions. Ind. Crops Prod. 177, 114496. doi: 10.1016/j.indcrop.2021.114496

[B19] Cahoon E. B. Schmid K. M. (2008). Metabolic engineering of the content and fatty acid composition of vegetable oils. Adv. Plant Biochem. Mol. Biol. 1, 161–200. doi: 10.1016/S1755-0408(07)01007-7

[B20] Canvin D. T. (1965). The effect of temperature on the oil content and fatty acid composition of the oils from several oil seed crops. Can. J. Bot. 43, 63–69. doi: 10.1139/b65-008

[B21] Celik Altunoglu Y. Unel N. M. Baloglu M. C. Ulu F. Can T. H. Cetinkaya R. (2018). Comparative identification and evolutionary relationship of fatty acid desaturase (FAD) genes in some oil crops: the sunflower model for evaluation of gene expression pattern under drought stress. Biotechnol. Biotechnol. Equip. 32, 846–857. doi: 10.1080/13102818.2018.1480421

[B22] Challinor A. J. Watson J. Lobell D. B. Howden S. M. Smith D. R. Chhetri N. (2014). A meta-analysis of crop yield under climate change and adaptation. Nat. Clim. Change 4, 287–291. doi: 10.1038/nclimate2153

[B23] Chaplin-Kramer R. O’Rourke M. E. Blitzer E. J. Kremen C. (2011). A meta-analysis of crop pest and natural enemy response to landscape complexity. Ecol. Lett. 14, 922–932. doi: 10.1111/j.1461-0248.2011.01642.x 21707902

[B24] Cheng C. Liu F. Sun X. Wang B. Liu J. Ni X.. (2022). Genomewide identification of FAD gene family and their contributions to the temperature stresses and mutualistic and parasitic fungi colonization responses in banana. Int. J. Biol. Macromol. 204, 661–676. doi: 10.1016/j.ijbiomac.2022.02.024 35181326

[B25] D’Angeli S. Altamura M. M. (2016). Unsaturated lipids change in olive tree drupe and seed during fruit development and in response to coldStress and acclimation. Int. J. Mol. Sci. 17, 1889. doi: 10.3390/ijms17111889 27845749 PMC5133888

[B26] De Borja Reis A. F. Rosso L. H. M. Davidson D. Kovács P. Purcell L. C. Below F. E.. (2021). Sulfur fertilization in soybean: A meta-analysis on yield and seed composition. Eur. J. Agron. 127, 126285. doi: 10.1016/j.eja.2021.126285

[B27] Delourme R. Falentin C. Huteau V. Clouet V. Horvais R. Gandon B.. (2006). Genetic control of oil content in oilseed rape (Brassica napus L.). Theor. Appl. Genet. 113, 1331–1345. doi: 10.1007/s00122-006-0386-z 16960716

[B28] Deng X. Scarth R. (1998). Temperature effects on fatty acid composition during development of low-linolenic oilseed rape (Brassica napus L.). J. Am. Oil Chem. Soc 75, 759–766. doi: 10.1007/s11746-998-0223-4

[B29] Di Rienzo J.A. Casanoves F. Balzarini M.G. Gonzalez L. Tablada M. Robledo C.W. (2008). InfoStat, versión 2008. Argentina: Grupo InfoStat, FCA, Universidad Nacional de Córdoba.

[B30] Dong C. J. Cao N. Zhang Z. G. Shang Q. M. (2016). Characterization of the fatty acid desaturase genes in cucumber: structure, phylogeny, and expression patterns. PloS One 11, e0149917. doi: 10.1371/journal.pone.0149917 26938877 PMC4777478

[B31] Dybing C. D. Zimmerman D. C. (1966). Fatty acid accumulation in maturing flaxseeds as influenced by environment. Plant Physiol. 41, 1465–1470. doi: 10.1104/pp.41.9.1465 16656425 PMC550555

[B32] Echarte M. M. Angeloni P. Jaimes F. Tognetti J. Izquierdo N. G. Valentinuz O.. (2010). Night temperature and intercepted solar radiation additively contribute to oleic acid percentage in sunflower oil. F. Crops Res. 119, 27–35. doi: 10.1016/j.fcr.2010.06.011

[B33] Falcone D. L. Ogas J. P. Somerville C. R. (2004). Regulation of membrane fatty acid composition by temperature in mutants of Arabidopsis with alterations in membrane lipid composition. BMC Plant Biol. 4, 1–15. doi: 10.1186/1471-2229-4-17 15377388 PMC524174

[B34] Fernández-Martínez J. M. Mancha M. Osorio J. Garcés R. (1997). Sunflower mutant containing high levels of palmitic acid in high oleic background. Euphytica 97, 113–116. doi: 10.1023/A:1003045726610

[B35] Fernández-Moya V. Martínez-Force E. Garcés R. (2002). Temperature effect on a high stearic acid sunflower mutant. Phytochemistry 59, 33–37. doi: 10.1016/S0031-9422(01)00406-X 11754941

[B36] Fiorani F. Schurr U. (2013). Future scenarios for plant phenotyping. Annu. Rev. Plant Biol. 64, 267–291. doi: 10.1146/annurev-arplant-050312-120137 23451789

[B37] Garcés R. Martínez-Force E. Salas J. J. Venegas-Calerón M. (2009). Current advances in sunflower oil and its applications. Lipid Technol. 21, 79–82. doi: 10.1002/lite.200900016

[B38] Garcés R. Sarmiento C. Mancha M. (1992). Temperature regulation of oleate desaturase in sunflower (Helianthus annuus L.) seeds. Planta 186, 461–465. doi: 10.1007/BF00195328 24186744

[B39] García-Inza G. P. Castro D. N. Hall A. J. Rousseaux M. C. (2014). Responses to temperature of fruit dry weight, oil concentration, and oil fatty acid composition in olive (Olea europaea L. var.’Arauco’). Eur. J. Agron. 54, 107–115. doi: 10.1016/j.eja.2013.12.005

[B40] García-Inza G. P. Castro D. N. Hall A. J. Rousseaux M. C. (2016). Opposite oleic acid responses to temperature in oils from the seed and mesocarp of the olive fruit. Eur. J. Agron. 76, 138–147. doi: 10.1016/j.eja.2016.03.003

[B41] García-Inza G. P. Hall A. J. Rousseaux M. C. (2018). Proportion of oleic acid in olive oil as influenced by the dimensions of the daily temperature oscillation. Sci. Hortic. 227, 305–312. doi: 10.1016/j.scienta.2017.09.030

[B42] Green A. G. (1986). Genetic control of polyunsaturated fatty acid biosynthesis in flax (Linum usitatissimum) seed oil. Theor. Appli. Genet. 72, 654–661. doi: 10.1007/BF00289004 24248076

[B43] Hall A. J. Richards R. A. (2013). Prognosis for genetic improvement of yield potential and water-limited yield of major grain crops. F. Crops Res. 143, 18–33. doi: 10.1016/j.fcr.2012.05.014

[B44] Izquierdo N. G. Aguirrezábal L. A. N. (2008). Genetic variability in the response of fatty acid composition to minimum night temperature during grain filling in sunflower. F. Crops Res. 106, 116–125. doi: 10.1016/j.fcr.2007.10.016

[B45] Izquierdo N. G. Aguirrezábal L. A. Andrade F. H. Cantarero M. G. (2006). Modeling the response of fatty acid composition to temperature in a traditional sunflower hybrid. Agron. J. 98, 451–461. doi: 10.2134/agronj2005.0083

[B46] Izquierdo N. G. Aguirrezábal L. A. N. Andrade F. H. Geroudet C. Valentinuz O. Iraola M. P. (2009). Intercepted solar radiation affects oil fatty acid composition in crop species. F. Crops Res. 114, 66–74. doi: 10.1016/j.fcr.2009.07.007

[B47] Izquierdo N. Aguirrezábal L. Andrade F. Pereyra V. (2002). Night temperature affects fatty acid composition in sunflower oil depending on the hybrid and the phenological stage. F. Crops Res. 77, 115–126. doi: 10.1016/S0378-4290(02)00060-6

[B48] Izquierdo N. G. Aguirrezábal L. A. N. Martínez-Force E. Garcés R. Paccapelo V. Andrade F.. (2013). Effect of growth temperature on the high stearic and high stearic-high oleic sunflower traits. Crop Pasture Sci. 64, 18–25. doi: 10.1071/CP12437

[B49] Izquierdo N. Benech-Arnold R. Batlla D. Belo R. G. Tognetti J. (2017). “Seed composition in oil crops: its impact on seed germination performance,” in Oilseed crops: yield and adaptations under environmental stress Chichester, West Sussex: Wiley-Blackwell, 34–51.

[B50] Kinney A. J. Knowlton S. (1998). “Designer oils: the high oleic acid soybean,” in Genetic modification in the food industry: A strategy for food quality improvement (Springer US, Boston, MA), 193–213.

[B51] Kobayashi K. Salam M. U. (2000). Comparing simulated and measured values using mean squared deviation and its components. Agron. J. 92, 345–352. doi: 10.2134/agronj2000.922345x

[B52] León A. J. Zambelli A. D. Reid R. J. Morata M. M. Kaspar M. Martínez-Force E.. (2013). Isolated mutated nucleotide sequences that encode a modified oleate desaturase sunflower protein, modified protein, methods and uses. WIPO Patent WO/2013/004280.

[B53] Mabhaudhi T. Chimonyo V. G. P. Hlahla S. Massawe F. Mayes S. Nhamo L.. (2019). Prospects of orphan crops in climate change. Planta 250, 695–708. doi: 10.1007/s00425-019-03129-y 30868238 PMC6667417

[B54] Mack L. Munz S. Capezzone F. Hofmann A. Piepho H. P. Claupein W.. (2018). Sowing date in Egypt affects chia seed yield and quality. Agron. J. 110, 2310–2321. doi: 10.2134/agronj2018.05.0324

[B55] Mafrica R. Piscopo A. De Bruno A. Poiana M. (2021). Effects of climate on fruit growth and development on olive oil quality in cultivar carolea. Agriculture 11, 147. doi: 10.3390/agriculture11020147

[B56] Martínez-Force E. Álvarez-Ortega R. Cantisán S. Garcés R. (1998). Fatty acid composition in developing high saturated sunflower (Helianthus annuus) seeds: maturation changes and temperature effect. J. Agric. Food Chem. 46, 3577–3582. doi: 10.1021/jf980276e

[B57] Martínez-Force E. Álvarez-Ortega R. Garcés R. (1999). Enzymatic characterisation of high-palmitic acid sunflower (Helianthus annuus L.) mutants. Planta 207, 533–538. doi: 10.1007/s004250050514

[B58] Martínez-Rivas J. M. Garcia-Diaz M. T. Mancha M. (2000). Temperature and oxygen regulation of microsomal oleate desaturase (FAD2) from sunflower. Biochem. Soc. Trans. 28, 890–992. doi: 10.1042/0300-5127:0280890 11171247

[B59] Martínez-Rivas J. M. Sperling P. Lühs W. Heinz E. (2001). Spatial and temporal regulation of three different microsomal oleate desaturase genes (FAD2) from normal-type and high-oleic varieties of sunflower (Helianthus annuus L.). Molec. Breed. 8, 159–168. doi: 10.1023/A: 1013324329322

[B60] Minervini M. Giuffrida M. V. Perata P. Tsaftaris S. (2017). Phenotiki: an open software and hardware platform for affordable and easy image based phenotyping of rosette-shaped plants. Plant J. 90, 204–216. doi: 10.1111/tpj.2017.90.issue-1 28066963

[B61] Muratorio A. (2003). Composición de ácidos grasos del aceite de girasol obtenido de semillas certificadas sembradas en distintas zonas de la República Argentina. Buenos Aires, Cosecha 2001–02.

[B62] Narayanan S. Zoong Lwe Z. S. Gandhi N. Welti R. Fallen B. Smith J. R.. (2020). Comparative lipidomic analysis reveals heat stress responses. Plants. 9 (4), 457. doi: 10.3390/plants9040457 32260392 PMC7238245

[B63] Ngidi A. Shimelis H. Chaplot V. Shamuyarira K. Figlan S. (2024). Biomass allocation and carbon storage in the major cereal crops: A meta-analysis. Crop Sci. doi: 10.1002/csc2.21294

[B64] Nicotra A. B. Atkin O. K. Bonser S. P. Davidson A. M. Finnegan E. J. Mathesius U.. (2010). Plant phenotypic plasticity in a changing climate. Trends Plant Sci. 15, 684–692. doi: 10.1016/j.tplants.2010.09.008 20970368

[B65] Osorio J. Fernández-Martínez J. Mancha M. Garcés R. (1995). Mutant sunflowers with high concentration of saturated fatty acids in the oil. Crop Sci. 35, 739–742. doi: 10.2135/cropsci1995.0011183X003500030016x

[B66] Peirone L. S. Pereyra Irujo G. A. Bolton A. Erreguerena I. Aguirrezábal L. A. (2018). Assessing the efficiency of phenotyping early traits in a greenhouse automated platform for predicting drought tolerance of soybean in the field. Front. Plant Sci. 9, 587. doi: 10.3389/fpls.2018.00587 29774042 PMC5943574

[B67] Pieruschka R. Schurr U. (2019). Plant phenotyping: past, present, and future. Plant Phenomics. 2019, 7507131. doi: 10.34133/2019/7507131 33313536 PMC7718630

[B68] Poorter H. Bühler J. van Dusschoten D. Climent J. Postma J. A. (2012). Pot size matters: a meta-analysis of the effects of rooting volume on plant growth. Funct. Plant Biol. 39, 839–850. doi: 10.1071/FP12049 32480834

[B69] Poorter H. Fiorani F. Pieruschka R. Wojciechowski T. van der Putten W. H. Kleyer M.. (2016). Pampered inside, pestered outside? Differences and similarities between plants growing in controlled conditions and in the field. New Phytol. 212, 838–855. doi: 10.1111/nph.2016.212.issue-4 27783423

[B70] Poorter H. Niinemets Ü. Walter A. Fiorani F. Schurr U. (2010). A method to construct dose–response curves for a wide range of environmental factors and plant traits by means of a meta-analysis of phenotypic data. J. Exp. Bot. 61, 2043–2055. doi: 10.1093/jxb/erp358 20048331

[B71] Poorter H. Yin X. Alyami N. Gibon Y. Pons T. L. (2022). MetaPhenomics: quantifying the many ways plants respond to their abiotic environment, using light intensity as an example. Plant Soil. 476, 421–454. doi: 10.1007/s11104-022-05391-8

[B72] Pushkova E. N. Povkhova L. V. Dvorianinova E. M. Novakovskiy R. O. Rozhmina T. A. Gryzunov A. A. (2024). Expression of FAD and SAD genes in developing seeds of flax varieties under different growth conditions. Plants 13, 956. doi: 10.3390/plants13070956 38611485 PMC11013676

[B73] Rebetzke G. J. Pantalone V. R. Burton J. W. Carver B. F. Wilson R. F. (1996). Phenotypic variation for saturated fatty acid content in soybean. Euphytica 91, 289–295. doi: 10.1007/BF00033090

[B74] Regitano Neto A. Miguel A.M.R.D.O. Mourad A. L. Henriques E. A. Alves R. M. V. (2016). Environmental effect on sunflower oil quality. Crop Breed. Appl. Biotechnol. 16, 197–204. doi: 10.1590/1984-70332016v16n3a30

[B75] Repository. doi: 10.6084/m9.figshare.24994631.v1

[B76] Rife C. L. Zeinali H. (2003). Cold tolerance in oilseed rape over varying acclimation durations. Crop Sci. 43, 96–100. doi: 10.2135/cropsci2003.9600

[B77] Rondanini D. P. del Pilar Vilariño M. Roberts M. E. Polosa M. A. Botto J. F. (2014). Physiological responses of spring rapeseed (Brassica napus) to red/far-red ratios and irradiance during pre-and post-flowering stages. Physiol. Plant 152, 784–794. doi: 10.1111/ppl.2014.152.issue-4 24814241

[B78] Scarth R. McVetty P. B. (1999). “Designer oil canola–a review of new food-grade Brassica oils with focus on high oleic, low linolenic types,” in Proceedings of the 10th International Rapeseed Congress, Canberra, Australia. 26–29.

[B79] Schulte L. R. Ballard T. Samarakoon T. Yao L. Vadlani P. Staggenborg S.. (2013). Increased growing temperature reduces content of polyunsaturated fatty acids in four oilseed crops. Ind. Crops Prod. 51, 212–219. doi: 10.1016/j.indcrop.2013.08.075

[B80] Secchi M. A. Fernandez J. A. Stamm M. J. Durrett T. Prasad P. V. Messina C. D.. (2023). Effects of heat and drought on canola (Brassica napus L.) yield, oil, and protein: A meta-analysis. F. Crops Res. 293, 108848. doi: 10.1016/j.fcr.2023.108848

[B81] Sigmaplot (2016). Windows (Chicago, Illinois: SPSS Inc.).

[B82] Soldatov K. I. (1976). “Chemical mutagenesis in sunflower breeding,” in Proc. 7th Int. Sunflower Conf., Krasnodar, USSR. International Sunflower Association., Vol. 27. 352–357.

[B83] Tardieu F. Parent B. (2017). Predictable ‘meta-mechanisms’ emerge from feedbacks between transpiration and plant growth and cannot be simply deduced from short-term mechanisms. Plant Cell Envir. 40, 846–857. doi: 10.1111/pce.12822 27569520

[B84] Tetreault H. M. Kawakami T. Ungerer M. C. (2016). Low temperature tolerance in the perennial sunflower Helianthus maximiliani. Am. Midland Nat. 175, 91–102. doi: 10.1674/amid-175-01-91-102.1

[B85] Tremolieres A. Dubacq J. P. Drapier D. (1982). Unsaturated fatty acid in maturing seeds of sunflower and rape: regulation by temperature and light intensity. Phytochemistry 21, 4145. doi: 10.1016/0031-9422(82)80011-3

[B86] Van K. McHale L. K. (2017). Meta-analyses of QTLs associated with protein and oil contents and compositions in soybean [Glycine max (L.) Merr.] seed. Int. J. Mol. Sci. 18, 1180. doi: 10.3390/ijms18061180 28587169 PMC5486003

[B87] Vuorinen A. L. Kalpio M. Linderborg K. M. Kortesniemi M. Lehto K. Niemi J.. (2014). Coordinate changes in gene expression and triacylglycerol composition in the developing seeds of oilseed rape (Brassica napus) and turnip rape (Brassica rapa). Food Chem. 145, 664–673. doi: 10.1016/j.foodchem.2013.08.108 24128529

[B88] Werteker M. Lorenz A. Johannes H. Berghofer E. Findlay C. S. (2010). Environmental and varietal influences on the fatty acid composition of rapeseed, soybeans and sunflowers. J. Agron.Crop Sci. 196, 20–27. doi: 10.1111/j.1439-037X.2009.00393.x

[B89] XYScan program (2010). Version 3.2.2.

[B90] Zanetti F. Angelini L. G. Berzuini S. Foschi L. Clemente C. Ferioli F.. (2022). Safflower (Carthamus tinctorius L.) a winter multipurpose oilseed crop for the Mediterranean region: Lesson learnt from on-farm trials. Ind. Crops Prod. 184, 115042. doi: 10.1016/j.indcrop.2022.115042

[B91] Zanetti F. Eynck C. Christou M. Krzyżaniak M. Righini D. Alexopoulou E.. (2017). Agronomic performance and seed quality attributes of Camelina (Camelina sativa L. crantz) in multi-environment trials across Europe and Canada. Ind. CropsProd. 107, 602–608. doi: 10.1016/j.indcrop.2017.06.022

[B92] Zemour K. Adda A. Labdelli A. Dellal A. Cerny M. Merah O. (2021). Effects of genotype and climatic conditions on the oil content and its fatty acids composition of Carthamus tinctorius L. seeds. J. Agron. 11, 2048. doi: 10.3390/agronomy11102048

[B93] Zhao X. Jiang H. Feng L. Qu Y. Teng W. Qiu L. (2019). Genome-wide association and transcriptional studies reveal novel genes for unsaturated fatty acid synthesis in a panel of soybean accessions. BMC genomics 20, 1–16. doi: 10.1016/j.fcr.2011.11.019 30665360 PMC6341525

[B94] Zuil S. G. Izquierdo N. G. Luján J. Cantarero M. Aguirrezábal L. A. N. (2012). Oil quality of maize and soybean genotypes with increased oleic acid percentage as affected by intercepted solar radiation and temperature. Field Crops Res. 127, 203–214. doi: 10.1016/j.fcr.2011.11.019

