# Supplementary material for: Meta-analysis unravels common responses of seed oil fatty acids to temperature for a wide set of genotypes of different plant species
Source: Front Plant Sci. 2024 Nov 15;15:1476311. doi: 10.3389/fpls.2024.1476311 (PMC11604464; doi:10.3389/fpls.2024.1476311)
Supplement: Supplementary file 1 [file Presentation1.pdf]

Supplementary Material

1 Supplementary Data

2 Supplementary Figures and Tables

2.1 Supplementary Figures

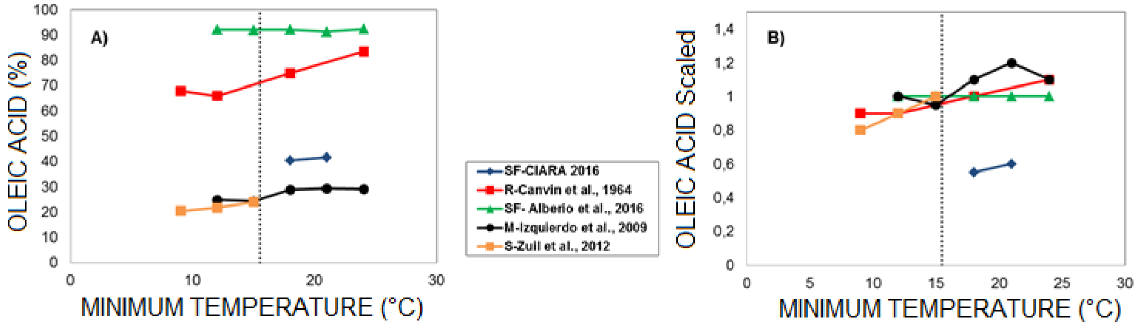

Supplementary Figure 1: **(A)** Oleic acid response to minimum temperature (Tmin, °C) for four different species measured in different experiments. **(B)** The same response after data normalization and interpolation.

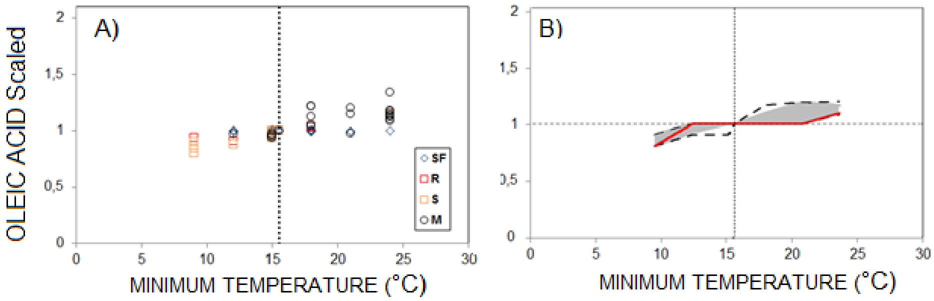

Supplementary Figure 2: **(A)** Literature data for ~ 45 oleic acid to minimum temperature observations from 4 experiments of a total of 4 species. Sunflower (○ SF), Rapeseed (□ R), Soybean (□ S), Maize (O M). **(B)** Reaction norm constructed from various segments of the data presented in A, indicated by the median value (bold red line), the interquartile range (the shaded area indicating the range between the 25th and the 75th percentile), and the 10th and 90th percentile (broken lines). In both charts: horizontal line represents the "non-response threshold" of the plotted data; vertical dotted line indicates the reference value of minimum temperature (15.5 °C) on the independent axis.

The direction and the magnitude of the reaction norm describe its shape. The extent of the phenotypic change accounted for dissimilar shapes for different genotypes across the same environmental range within a species (e.g. Vitasse et al., 2010) and among different species with the same trait and environmental range (e.g. Schou et al., 2017). Certain traits might not exhibit phenotypic plasticity, in which case the reaction norm would be flat, whereas for plastic traits, the slope of the reaction norm was nonzero. The direction and the magnitude of the reaction norm were represented by the

bold red line (Supplementary Figure 2 B) and the extent of the phenotypic change was represented by the shaded zone and dotted lines (Supplementary Figure 2 B).

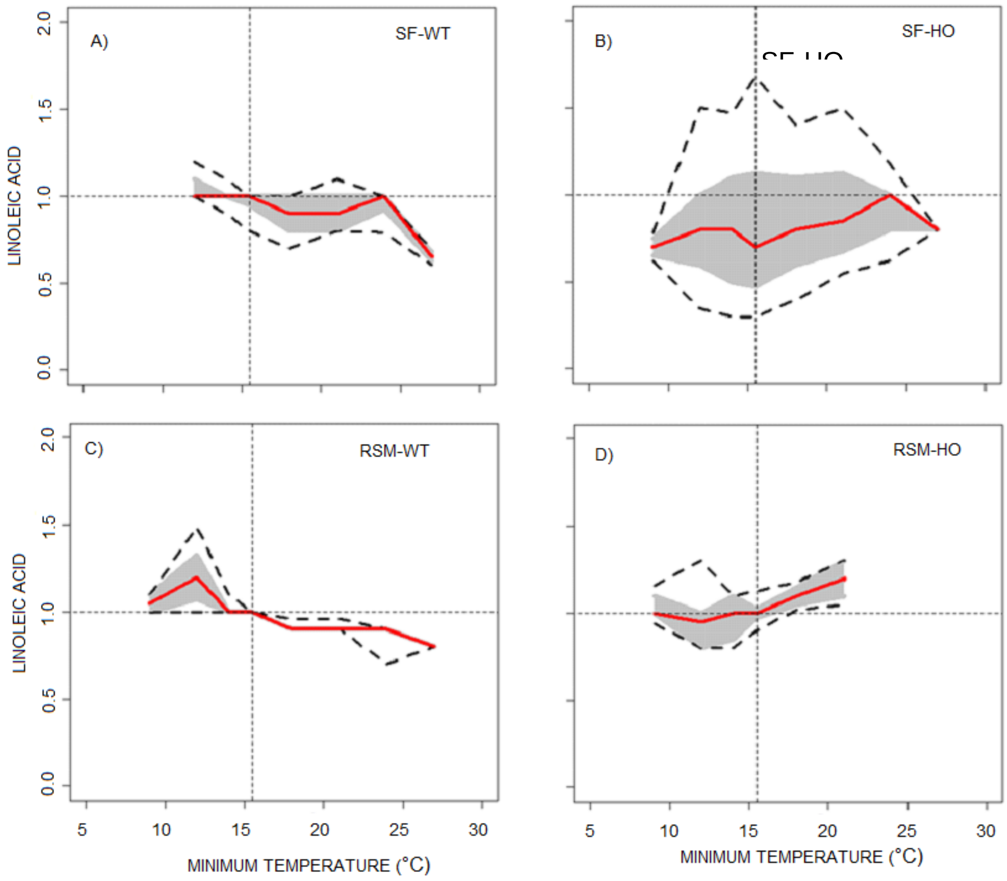

Supplementary Figure 3: Reaction norms of the linoleic acid to minimum temperature of SF and RSM divided in WT and HO. Median value (bold red line), the interquartile range (gray area), and the 10th and 90th percentile (broken lines).Horizontal line represents the "non-response threshold" of the plotted data; vertical dotted line indicates the reference value of minimum temperature (15.5 °C) on the independent axis.

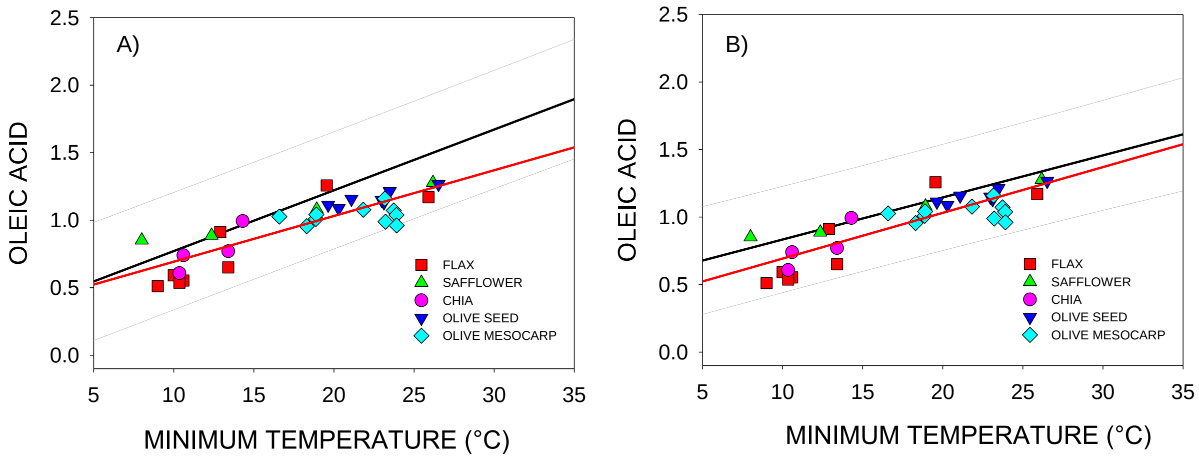

Supplementary Figure 4: Assessment of oleic acid response of **(A)** the WT-Model and **(B)** RSM-WT-Model using independent species (DataSet C). Bold/black lines corresponded to the regression lines of the WT or RSM-WT models. Grey lines correspond to the prediction intervals of the WT and RSM-WT models. Data were superimposed on WT-Model and on RSM-WT-Model. Bold/red lines corresponded to the regression lines of set of independent species. Symbols correspond to: ■ Flax, ▲ Safflower, ● Chia, ▼ Olive seed, ◇ Olive mesocarp.

2.2. Supplementary Tables

Supplementary Table 1: Comparison of ordinates to the origin and slopes between the established models and a set of genotypes carrying one mutation different from the HO and double mutations.

		Palmitic acid	Stearic acid	Oleic acid	Linoleic acid	Linolenic acid
WT-	Ordinate	0.05	0.4	-0.26	0.004	-1.04**
MODEL	Slope	-0.001	-0.019	0.01	-0.002	0.02
HO-	Ordinate	-0.07	-0.09	-0.002	0.14	-11.87***
MODEL	Slope	0.008	0.007	-0.0003	0.004	-0.06

* Asterisks indicate significant differences between the established models and a set of genotypes carrying one mutation different from the HO and double mutations.

Supplementary Table 2: Comparison of ordinates to the origin and slopes between the established models and a set of independent species selected for validation.

		Palmitic acid	Stearic acid	Oleic acid	Linoleic acid	Linolenic acid
GENERAL MODEL	Ordinate	0.84	0.06	0.12	0.07*	0.33
	Slope	0.61	0.02*	0.81	0.01*	0.55
WT-MODEL	Ordinate	0.87	0.24	0.8	$< 8.15e^{-06}$***	0.26
	Slope	0.96	0.11	0.11	0.001**	0.7
HO-MODEL	Ordinate	0.87	0.01*	$< 2e^{-16}$***	0.0001***	0.95
	Slope	0.96	0.003**	$< 2e^{-16}$***	$< 2.96e^{-05}$***	0.9
SF-MODEL	Ordinate	0.82	0.002**	0.53	0.007*	
	Slope	0.6	0.0003***	0.54	0.008**	
RSM-MODEL	Ordinate	0.59	0.75	$< 7.99e^{-14}$***	0.12	
	Slope	0.68	0.58	$< 5.47e^{-10}$***	0.82	
RSM-MODEL (WT)	Ordinate	0.58	0.13	0.17	0.0003***	0.26
	Slope	0.71	0.06	0.69	0.04*	0.7

* Asterisks indicate significant differences between the established models and a set of independent species selected for validation.
